# *MicrobiomeExplorer*: an R package for the analysis and visualization of microbial communities

**DOI:** 10.1093/bioinformatics/btaa838

**Published:** 2020-10-30

**Authors:** Janina Reeder, Mo Huang, Joshua S Kaminker, Joseph N Paulson

**Affiliations:** Department of OMNI Bioinformatics, Genentech, Inc, South San Francisco, CA 94080, USA; Department of Statistics, The Wharton School, University of Pennsylvania, Philadelphia, PA 19104, USA; Department of Biostatistics, Genentech, Inc, South San Francisco, CA 94080, USA; Department of OMNI Bioinformatics, Genentech, Inc, South San Francisco, CA 94080, USA; Department of Biostatistics, Genentech, Inc, South San Francisco, CA 94080, USA

## Abstract

**Summary:**

We developed the *MicrobiomeExplorer* R package to facilitate the analysis and visualization of microbial communities. The *MicrobiomeExplorer* R package allows a user to perform typical microbiome analytic workflows and visualize their results, either through the command line or an interactive Shiny application included with the package. In addition to applying common analytical workflows, the application enables automated analysis report generation.

**Availability and implementation:**

Available at https://github.com/zoecastillo/microbiomeExplorer.

**Supplementary information:**

Supplementary data are available at *Bioinformatics* online.

## 1 Introduction

High-throughput DNA-sequencing technologies, including targeted marker-gene sequencing, are providing unprecedented insight into the composition and population structure of microbial communities. Researchers are rapidly applying these new technologies in a variety of clinical settings, to understand the structure and function of bacteria ranging varied communities (Chopyk *et al.*, 2017). Although it may seem that DNA sequencing should reduce many scientific questions to simply counting the representative sequence of the organism, there are subtle but significant challenges associated with sampling, counting, and representational statistics. As a consequence, many microbiome-specific analytical pipelines and workflows have been developed.

Although tools and packages exist to answer many of the questions asked of this exciting new data type, analyses remain inaccessible to researchers without the appropriate skill-set. The *MicrobiomeExplorer* R package allows users to analyze microbiome data either from the R command line or through an interactive Shiny application. It aims to address the needs of computational scientists by combining different analysis methods in one package, and also the needs of bench scientists with a limited coding background. In addition, it delivers a set of powerful and well-designed interactive visualizations based on the *plotly* package (Sievert, 2020). This distinguishes it from previous approaches such as *phyloseq* and *metaviz* which are command line or do not contain all the visualization capabilities featured (Wagner *et al.*, 2018).

## 2 *Microbiomeexplorer* R package

As input, *MicrobiomeExplorer* accepts several different input formats such as raw count tables, BIOM-formatted files, and MRexperiment objects from the *metagenomeSeq* R package (McMurdie and Paulson, 2016; Paulson *et al.*, 2013). Raw counts and BIOM files can be annotated using tabular phenotype information on the samples as well as a feature table describing the taxonomic features identified.

**Fig. 1.**
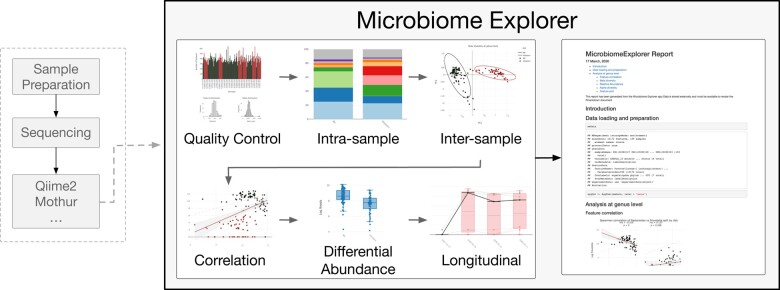
Flow diagram for the *MicrobiomeExplorer* R package. Following sample preparation, sequencing and post-processing of reads by tools like Qiime2 and Mothur, counts, taxonomy and associated metadata can be uploaded into the *MicrobiomeExplorer* explorer. Modules include (i)quality control, (ii) intra-samples, (iii) inter-sample/intra-feature, (iv) correlation, (v) differential abundance and (vi) longitudinal analyses. Downstream of this, the *MicrobiomeExplorer* tool allows for downloadable reports

For data pre-processing, users can perform filtering of poor quality samples through exploratory data analysis of the sequencing depth and number of features observed. Normalization is enabled to calculate proportions or by using cumulative sum scaling. Subsetting of samples to exclude certain phenotypes or modifying the phenotype data by combining different columns or adjusting the data types is available. Additionally, provided feature data can be updated by rolling down information from higher taxonomy levels if an operational taxonomic unit (OTU) cannot be uniquely identified (Fig. 1).

### 2.1 Analysis workflow and reproducibility reporting

Organization of the analysis workflow consists of multiple analysis types separated into: overview and pre-processing, intra-, inter-sample, intra-feature, feature correlation, differential abundance and longitudinal analyses.

The first step of any analysis within the application is to pre-process the data. Initial visualizations help in assessing the quality and characteristics of sequencing depth and feature presence.

The second step of an analysis is to choose a taxonomy level and aggregate data at the chosen level for downstream analyses.

Intra-sample analysis contains functions that aid in the investigation of the microbial composition within a sample or a group of samples. The user can explore the relative abundance, feature abundance or alpha diversity. Alpha diversity is implemented through the *vegan* package and includes diversity metrics such as diversity (Oksanen *et al.*, 2007).

Inter-sample analyses focus on differences between samples or groups of samples. This is accomplished through the use of heatmaps of variable or differentially abundant OTUs, or features and beta diversity calculations. Beta diversity distance metrics vary within the package and include Bray–Curtis. Within an analysis session of the application, computed distance matrices are cached for improved efficiency. Color annotations can be added for higher taxonomy levels or specific phenotypes.

Feature correlation analysis modules provide scatterplots of abundance between features and correlation statistics, both of which can be faceted by phenotypes. A test for significance within each facet can be performed. Phenotype correlation is similar, except it analyzes the relationship between a feature and a continuous phenotype.

Differential abundance testing can be performed using *DESeq2*, Kruskal–Wallis, *limma* or a zero-inflated log normal model. *DESeq2* and *limma* are widely used methods for comparisons in microarray and RNA-sequencing data that have been adapted for microbiome data. Kruskal–Wallis is a non-parametric test for any differences in distribution between groups. The zero-inflated log normal model is implemented in the *metagenomeSeq* package to account for zero-inflation in microbiome data.

Longitudinal analysis provides the option to visualize feature abundances across specific levels of a phenotype, such as dates or tissue types. Optionally, a separate phenotype can be used to link a common data point across the different levels with lines.

A key feature includes the ability to report select analyses and export them in HTML, PDF, Word document or Powerpoint with R markdown code for reproducibility and adjustments.

## 3 Conclusion

*MicrobiomeExplorer*, available at https://github.com/zoecastillo/microbiomeExplorer, is an R package specifically meant to help facilitate the analysis and visualization of microbial communities from marker-gene surveys or whole metagenomic shotgun sequencing. The tool provides a user-friendly interactive interface through Shiny with downloadable R Markdown scripts and reports.

## Supplementary Material

btaa838_Supplementary_Data
