# Supplementary material for: MicrobiomeExplorer: an R package for the analysis and visualization of microbial communities
Source: Bioinformatics. 2020 Oct 30;37(9):1317–8. doi: 10.1093/bioinformatics/btaa838 (PMC8193707; doi:10.1093/bioinformatics/btaa838)
Supplement: btaa838_Supplementary_Data [file btaa838_supplementary_data.html]

Using Microbiome Explorer application to analyze amplicon sequencing data


# Using Microbiome Explorer application to analyze amplicon sequencing data

```
library(knitr)
library(rmarkdown)
library(microbiomeExplorer)
library(magrittr)
library(metagenomeSeq)
library(shiny)

doctype <- opts_knit$get("rmarkdown.pandoc.to")
```

## Introduction

The microbiomeExplorer package and the Shiny application contained with it provides methods and visualizations to explore the results of 16S rRNA amplicon sequencing experiment. The analyses can be performed entirely via the R command line, but the primary intent of the package is to enable access to many of these analyses to non-computational users through the Shiny user interface.

The interface is launched through the R environment by calling `MicrobiomeExplorerApp()` after loading the package or made available via deployment on a Shiny server. This document is intended to give an introduction of how to use the Shiny application with the addition of R code chunks that a command line user would use to produce the same results.

```
runMicrobiomeExplorer()
```

## Data upload

#### Microbiome Explorer accepts several different data upload formats

- MRexperiment-class objects stored as RDATA or RDS files
- The Biological Observation Matrix (BIOM) formattes files produced by any program including qiime2 or mothur
- Raw counts files

A counts file is required and can be uploaded in delimited form (csv, tsv). The required format is such that each sample is a column and each unique feature is a row in the data set.

A delimited phenotype file can be linked to the counts data. The required format is such that each sample is a row with the names of the rows in the phenotype data corresponding to the names of the columns in the count data. Appending several phenotype files by subsequent uploads is possible. If no phenotype file is given, the names of the columns of the count data are used as the phenotype data.

A feature data file must be provided if aggregation to a particular phylogenetic level is desired. Each unique feature is a row which must correspond to the ones in the counts data. Each column is a taxonomy level.

If the feature data file is omitted, analysis can only be done at the raw counts level. An MRexperiment-class object from the the `metagenomeSeq` package already combines these different data files into one data structure based on an extended eSet-class.

#### Uploading data into the application

The mouseData example data set is included with the `metagenomeSeq` package on which `microbiomeExplorer` depends. The easiest way to make this data available for the application is to store it as an RDS object on your file system. Then, you can open it via the “Browse” button under *Upload Feature Count Data*.

```
data("mouseData", package = "metagenomeSeq")
meData <- filterMEData(mouseData,minpresence = 1, minfeats = 2, minreads = 2)
```

## Data QC

Before starting an analysis, it is recommend to review the results of the sequencing experiment and perform quality control. Some samples may have lower number of features than expected or an overall low number of reads. There are several ways to filter samples considered not useful for downstream analyses. Multiple QC plots can be generated, including those showing the number of unique features in each sample as a barplot or in a scatterplot against the number of reads. These can be colored by specific phenotypes stored in the pData slot of the MRexperiment. In addition, histograms show the overall distribution of feature and read frequencies.

```
makeQCPlot(meData, col_by = "diet",
       log = "none",
       filter_feat = 101,
       filter_read = 511,
       allowWebGL = FALSE)
```

```
plotlySampleBarplot(meData,
                    plotTitle = "Unique features per sample",
                    col_by = "diet")
```

## Data Filtering and Subsetting

Within the application, three different sliders can be used to adjust quantitave restrictions on the data. The user can require a feature to be present in a minimum number of samples, and they can require a sample to have a minimum number of features or reads. Subsetting via a specific phenotype is also possible. This provides the option to exclude certain samples or limit the analysis to a subset of the data.

```
meData <- filterMEData(mouseData,minpresence = 1, minfeats = 100, minreads = 500)
```

## Normalization

Normalization allows for the user to account for library size differences before analysis. Certain app features are restricted if not done (e.g. percentage is unavailable if not normalized). Differential abundance testing also requires normalization, which we perform silently if the user does not choose to do so. The two available methods included in the package are based on either calculating proportions or by using cumulative sum scaling (CSS), Paulson, et al. Nat Meth 2013.

```
meData <- normalizeData(meData,norm_method = "Proportion")
```

## Phenotable alteration

A new phenotable can easily be added to the MRexperiment. The application offers users similar options by allowing the extension or replacement of the pheno data via the load & filter section or by small in-app modifications of the table via the phenotype section. Here, the user can combine two columns to create a new phenotype based on a concatenation of the data. This new phenotype can then be used in the analysis. We provide the option to adjust data types, such as converting a declared numeric variable a factor, etc. This is typically not necessary, but may be useful or more appropriate, eg. in an example where cages are numeric. Finally, the user can simplify phenodata by selecting only necesssary columns. Changes made need to be saved in order to update the underlying data structure for analysis.

```
new_pheno <- interaction(pData(meData)[,c("mouseID","relativeTime")])
mutatedRows <- row.names(pData(meData))
mutatedData <- dplyr::mutate(pData(meData), "mouse_time" = new_pheno)
row.names(mutatedData) <- mutatedRows
meData <- addPhenoData(meData,mutatedData)
```

## Feature table alteration

The feature table provides an overview of the taxonomy annotation associated with each unique feature. Depending on the results, you might see many empty cells at specific taxonomy levels, eg. species or strain. These empty cells indicate that a feature could not be successfully assigned to a specific genus or strain. These can be left as are, marked as unknown or roll down the taxonomy. In this case, the most specific taxonomy annotation available for each feature is pushed down to more specific levels, e.g. “unknown\_Firmicutes”. The advantage of this method lies in the way analyses are done on the data. The user chooses a specific taxonomy level to analyze and all available feature counts are summarized down to unique features at this particular level. Two features with missing annotation cannot be distinguished, but an “unknown\_Firmicutes” can be distinguished from an “unknown\_Clostridiales”. Any changes need to first be assigned and then saved in order to update the underlying data structure for analysis.

```
bufcolnames <- names(fData(meData))
df <- as.data.frame(t(apply(fData(meData),1, rollDownFeatures)))
names(df) <- bufcolnames
meData <- addFeatData(meData,df)
```

# Analysis

The analysis workflow within the application is split into five different sections: intra sample, inter sample, correlation, differential and longitudinal. Each section will be described below. All visualizations are implemented using the plotly R package which provides basic interactivity, including zooming or panning via its modebar. In addition, the user can export the plot in its current state (i.e. showing specific user interactions) as a svg file using the camera icon of the modebar.

## Aggregation

Before any analysis is possible, the user needs to aggregate the data down to a specific feature level. The available levels can be restricted via the code in global.R. Once this is completed, the analysis sections will be enable for use. Alternatively, the user can choose to add an analysis to a report by clicking the “Report” button which will recreate the visualizations as described in the reports section of this document.

```
aggDat <- aggFeatures(meData, level = "genus")
```

## Intra-Sample Analysis

Intra-sample analysis contains functions focus on investigating the microbial composition within a sample or a group of samples. Different functions are available to visualize the relative abundance of top features, the abundance of a specific feature as well as the alpha diversity within the sample. Within the application, one common set of input elements is used to generate all visualization.

### Relative Abundance

Relative abundance shows the most abundant feature in a barplot summarized by a user-defined variable across the x-asis. In addition, the user can choose to facet by phenotypes, adjust the number of features to show, switch between showing total numbers (Reads) and normalized value (if normalized), and modify the overall plot width. Clicking on a specific feature in the plot, automatically opens a feature abundance plot for this feature.

```
plotAbundance(aggDat,
              level = "genus",
              x_var = "diet",
              facet1 = NULL,
              facet2 = NULL,
              ind = 1:10,
              plotTitle = "Top 10 feature percentage at genus level",
              ylab = "Percentage")
```

### Feature abundance

The feature abundance plot shows the individual abundance of a specific feature either as a boxplot or a categorical scatterplot depending on the x-axis variable chosen. The user can choose to employ a \(\text{log}\_2\) scale, define plot width and decide wether to show individual sample points or not. Feature plots can be opened by selecting a specific feature in the input section or by clicking on a feature in the relative abundance plot.

```
plotSingleFeature(aggDat,
            x_var = "diet",
            ind = 1:10,
            plotTitle = "Percentage of Enterococcus",
            facet1 = NULL,
            facet2 = NULL,
            feature = "Enterococcus",
            ylab = "Percentage",
            log = TRUE,
            showPoints = TRUE)
```

### Alpha Diversity

Alpha diversity is a measure of the complexity or diversity within a particular sample, eg. habitat or area. Alpha diversity is computed by functions in the vegan package and is visualized as a boxplot using the same input definitions by feature and relative abundance. The user can choose to color and thus split the boxes by a phenotype and set the overall plot width. Multiple diversity measures are offered with Shannon diversity provided as the default. We suggest users read up on the various measures and understand the differences in interpretation and nuances.

Shannon diversity in particular measures how evenly the microbes are distributed in a sample and is defined by the following relationship where \(p\_i\), is the proportion of an individual feature: \(H = -\sum\_{i=1}^T p\_i \ln p\_i\).

```
plotAlpha(aggDat,
          level = "genus",
          index = "shannon",
          x_var = "diet",
          facet1 = NULL,
          facet2 = NULL,
          col_by = "mouseID",
          plotTitle = "Shannon diversity index at genus level")
```

## Inter Sample Analysis

Inter-sample analyses focus on differences between samples or groups of samples via feature heatmaps and beta diversity calculations. Please note that these functions can be computationally intensive if there are many samples and a low aggregation level is chosen.

### Beta Diversity

Beta diversities are the measures of the complexity of communities between samples, as compared to within a sample (alpha diversity). Calculating beta diversity first requires the computation of a pairwise distance or similarity matrix. The user can select between different measures offered via the vegan package with Bray being the suggested default selection for microbiome analysis. We suggest users read up on the various measures and understand the differences in interpretation and nuances.

Principal component analysis, a dimension reduction method, is subsequently performed on the chosen distance matrix and visualized in a scatter plot. The user has the option to choose the principal components to display, add coloring and confidence ellipses based on a phenotype, define the shape based on a phenotype and adjust both the point size as well as the overall plot width.

PERMANOVA (permutational multivariate analysis of variance), from the vegan package is offered via the application (command line users will need to run this function independently and pass the results to the plotting function). Conceptually, a PERMANOVA analysis lets the user statistically determine if the centroids of a dissimilarity or distance matrix differ between groups of samples. Optionally, the user can select an phenotype as well as a strata variable with the results being shown, both within the visualization as well as in a table below it.

```
distMat <- computeDistMat(aggDat, "bray")
pcaVals <- calculatePCAs(distMat, 
                         c("PC1", "PC2"))
plotBeta(aggDat,
         dist_method = "bray",
         pcas = pcaVals,
         dim = c("PC1", "PC2"),
         col_by = "diet",
         shape_by = NULL,
         plotTitle = "Bray-Curtis diversity at genus level",
         pt_size = "6",
         plotText = "R2: 0.478; Pr(>F): 0.002",
         confInterval = 0.95,
         allowWebGL = FALSE)
```

### Heatmap

The heatmap offers another view on differences and similarities between the samples in a dataset. The user can either choose specific features or show the top 50 features sorted either by variance, Fano factor or median absolute deviation (MAD). The visualization is done with heatmaply which in turns relies on plotly to render the heatmap. The same options to interact with the plot are thus available. Once rendered, the user can change the number of features to include, turn of log scale, and add annotation to both rows (phenotypes) and columns (higher taxonomy levels) of the heatmap. It is recommended to not use the heatmap functionality in datasets with many samples (5000+) as this can be quite slow to render.

```
plotHeatmap(aggDat,
            features = NULL,
            log = TRUE,
            sort_by = "Variance",
            nfeat = 50,
            col_by = c("diet"),
            row_by = "",
            plotTitle = "Top 50 features sorted by Variance at genus level")
#> Registered S3 method overwritten by 'dendextend':
#>   method     from 
#>   rev.hclust vegan
#> Registered S3 method overwritten by 'gclus':
#>   method         from 
#>   reorder.hclust vegan
#> Registered S3 method overwritten by 'seriation':
#>   method         from 
#>   reorder.hclust gclus
```

## Correlation

Correlation allows the user to visualize the relationship between either two features or a feature and a numeric phenotype in a scatterplot enhanced with a linear regression statistic. Faceting and/or coloring by phenotypes is available in both correlation plots. The user is asked to choose between three different methods to aid in the evaluation of the association: Spearman (default), Pearson or Kendall.

```
cf <- corrFeature(aggDat,
                 feat1 = "Bacteroides",
                 feat2 = "Prevotella",
                 log = TRUE,
                 facet1 = "diet",
                 facet2 = NULL,
                 method = "spearman",
                 plotTitle = "Spearman correlation of Bacteroides vs Prevotella split by diet",
                 col_by = "status",
                 allowWebGL = FALSE)
```

## Differential abundance

Differential abundance (DA) analysis is focused on testing the null hypothesis that the mean or mean ranks between groups are the same for a specific feature. DA analysis can help detect changes in feature abundance across two or more different levels of a phenotype. Four different methods can be chosen via the application: DESeq2, Kruskal-Wallis, limma, or a zero-inflated log normal model. DESeq2 and limma are widely used methods for comparisons in microarray and RNA-sequencing data which can easily be adapted for microbiome data. Kruskal-Wallis is a non-parametric test for any differences in distribution between groups. The zero-inflated log normal model is implemented in the metagenomeSeq package to account for zero-inflation in microbiome data. Typically, DESeq2 would is used with small (<=25) sample sizes. The results will be displayed in an interactive table (DT) within the application and the user can open feature plots showing the specific levels by clicking on a row of interest.

```
diffResults <- runDiffTest(aggDat,
                        level = "genus",
                        phenotype = "diet",
                        phenolevels = c("BK", "Western"),
                        method = "DESeq2")

kable(head(diffResults))
```

| genus | baseMean | log2FoldChange | lfcSE | stat | pvalue | padj |
| --- | --- | --- | --- | --- | --- | --- |
| unknown\_Lachnospiraceae | 545.087 | -0.436 | 0.106 | -4.107 | 0 | 0 |
| Bacteroides | 377.256 | -1.259 | 0.142 | -8.855 | 0 | 0 |
| Prevotella | 359.280 | -6.317 | 0.311 | -20.284 | 0 | 0 |
| LachnospiraceaeIncertaeSedis | 200.745 | 1.148 | 0.062 | 18.600 | 0 | 0 |
| ErysipelotrichaceaeIncertaeSedis | 119.196 | 3.553 | 0.147 | 24.247 | 0 | 0 |
| Enterococcus | 101.853 | 8.351 | 0.427 | 19.543 | 0 | 0 |

## Longitudinal

Longitudinal analysis allows the user to generate feature plots with more control over the data shown within the plot. For a specific feature, the user can choose a phenotype and specific levels of that phenotype to show in the plot. The chosen order of the levels will be kept within the visualization which allows sorting by specific dates or tissues among other things. If desired and available, the user can choose a specific phenotype to summarize on which will then be connected by lines across the different levels. The resulting visualization is interactive and the user can then select and color specific IDs within the plot.

```
plotLongFeature(aggDat,
                x_var = "date",
                id_var = "mouseID",
                plotTitle = "Abundance of Prevotella",
                feature = "Prevotella",
                ylab = "Reads",
                log = TRUE,
                x_levels = c("2007-12-11","2008-01-21","2008-02-11","2008-02-25"))
```

# Reports

Once an analysis is complete, a user might like to share the results with collaborators or download them for further analysis beyond the functionality provided via the Microbiome Explorer. To do this, we are providing the option to include any part of the analysis in a report which is fully reproducible outside of the application.

### Report Settings

In the report settings, the user can choose file name and add a project title, authors as well as any other text to be included in the output. Four different output formats are available by default. Please note that report generation does rely on the availability of external programs such as pandoc and latex (pdf). The available output formats can be restricted in global.R.

In addition to the basic settings, the user can also review any analysis made and choose which parts should be included in the report and which shouldn’t. For each analysis element, the relevant R code is shown next to an icon illustrating the type of analysis. This is intended to help users without a computational background to identify which section is related to which part of their analysis. Those steps that are essential such as data loading or aggregation cannot be deselected.

## Report Generation

In order to obtain the report, the user first clicks the “Generate” button. Then, the relevant R code collected during the analysis anytime the user clicks the “Report” button is written to a temporary file. The temporary R file is then knitted into an Rmd document using knitr::spin and subsequently rendered to the desired output format(s) with rmarkdown::render. R code chunks are enhanced with basic parameters to optimize the sizing of figures. This works reasonably well for HTML output. In order to produce well-designed output in other formats, more editing of the Rmarkdown document is recommended.

During the process any analysis is repeated for each output format while calling the render function. In future versions of the application, we are planning to do this as a background process with the results emailed to users. For now, the user needs to patiently wait until the render process has completed and then download the results by clicking “EXPORT”. They will then obtain a zip folder which holds both the Rmd document as well as any output formats specified. The Rmd document can be edited and re-rendered outside of the application. The only modification required is to adjust the path to the input data which is unknown to the application.
